# Supplementary material for: Topical diclofenac vs placebo for the treatment of chronic Achilles tendinopathy: A randomized controlled clinical trial
Source: PLoS One. 2021 Mar 4;16(3):e0247663. doi: 10.1371/journal.pone.0247663 (PMC7932128; doi:10.1371/journal.pone.0247663)
Supplement: S1 Table — (DOCX) [file pone.0247663.s002.docx]

**Table S1. Baseline comparisons across sex groups**

| **Outcome** | **Men** | **Women** | **Adjusted p value** |
| --- | --- | --- | --- |
| **VISA-A^1^** | 63 (12) | 55 (18) | 0.11 |
| **Pain with activity^2^** | 3 (3) | 4 (1) | 0.19 |
| **Pain at rest^2^** | 1 (2) | 1 (3) | 0.24 |
| **Pain pressure threshold^1^** | 260 (180) | 170 (130) | 0.10 |
| **Tendon stiffness^1^** | 820 (130) | 740 (130) | 0.06 |

^1^ Mean (standard deviation) ^2^ Median (interquartile range)

There is one missing value for the “Pain Pressure Threshold” outcome. For all the ordinal outcomes (Pain Ratings for Rest and Activity), we conducted Mann-Whitney U tests. The null hypothesis for this non-parametric test is that the distributions of the two samples (males and females, here) are the same, while the alternative hypothesis is that the there is a location shift (one distribution has a higher median than the other). This is different from the parametric two-sample t-test, which is testing for the equality of means. For the remaining outcomes, we conducted two-sample t-tests.

The results of the tests are shown, with a multiple testing adjustment of the p-values using the Holm-Bonferroni Correction. None of the tests were statistically significant (at 5% significance level), suggesting that there aren’t significant differences in mean/median between males and females for each of the 5 outcomes at baseline.

The mean (or median for the Pain Ratings) and standard deviation (or interquartile range for Pain Ratings) for each of the sex groups is also shown. We do see that the measures of central tendency for men are generally higher than that for women (except for the Pain Ratings). Given the results of the tests above, there appears to be weak evidence of differences between the sex groups for all five outcomes.
