# Supplementary material for: Topical diclofenac vs placebo for the treatment of chronic Achilles tendinopathy: A randomized controlled clinical trial
Source: PLoS One. 2021 Mar 4;16(3):e0247663. doi: 10.1371/journal.pone.0247663 (PMC7932128; doi:10.1371/journal.pone.0247663)
Supplement: S1 File — (PDF) [file pone.0247663.s004.pdf]

Topical diclofenac as an adjunct to rehabilitation of chronic Achilles tendinopathy: a randomized controlled trial

Study Investigator(s)

Principal Investigator: Dr. Alexander Scott
Co-Investigators:
Erin Bussin, MSc
Dr. Jim Bovard
Dr. Brian Cairns
Dr. Tommy Gerschman
Dr. Jason Crookham

Additional study team member:

Dr. Michael Fredericson

Study Summary

**Study Purpose**	To evaluate the effect of topical diclofenac on clinical outcomes of chronic Achilles tendinopathy (CAT).
**Study Design**	Placebo controlled trial with random allocation.
**Aims**	**Primary:** To determine whether topical diclofenac affects clinical outcome after 4 weeks of treatment (VISA-A score).<br>**Secondary:** To determine if topical diclofenac changes pain, pain threshold, or mechanical properties of the Achilles tendon in subjects with chronic Achilles tendinopathy.
**Hypothesis**	Individuals treated with topical diclofenac will have a greater improvement in VISA-A score at 4 weeks than those treated with placebo gel.
**Sample Size**	100 subjects
**Study Location**	Vancouver, Alan McGavin Sports Medicine UBC, and Dr. Bovard's practice (1200 Lonsdale Ave, North Vancouver).
**Main Inclusion Criteria**	**Study Sample:** Male and female subjects aged 19 years and older; fluent in English; subject diagnosed with Achilles tendinopathy (unilateral or bilateral; midportion or insertional) by a health care professional; symptoms for 3 months or more; subjects who are able to give informed consent; VISA-A score less than 80; Subjects who are engaged in an active rehabilitation program prescribed by an exercise specialist.
**Main Exclusion Criteria**	**Study Sample:** Male and female subjects aged 18 years and younger; subjects with a BMI greater than 30.0; subjects with previous Achilles tendon rupture (complete or partial); subjects diagnosed with chronic pain syndrome, diabetes, or systemic inflammatory disease; subjects with a history of gastric ulcers, kidney disease or unstable hypertension; subjects with symptomatic osteoarthritis of the spine or lower extremities; subjects who have received corticosteroid injections in the Achilles region; subjects who take non-steroidal anti-inflammatory medication regularly; subjects who have been prescribed anticoagulants or fluroquinolones within the past 5 months; subjects with allergies to diclofenac or placebo gel; subjects who are unable to give informed consent.
**Intervention**	Topical 10% diclofenac, 1g massaged into tendon TID x 4 weeks vs placebo.
**Primary Outcome**	VISA-A score (4 weeks)
**Secondary Outcomes**	1. Pain (Numeric Pain Rating Scale, 0 - 10)

i. Average pain during all tendon loading activities over last week
ii. Average pain during rehabilitative exercise over last week
iii. Average pain at rest during last week
iv. Current resting pain

2. Patient-reported change in symptoms (7 point scale from substantially better to substantially worse)
3. Pressure pain threshold (N) and tendon stiffness (N/m)

1. Introduction

Chronic Achilles tendinopathy, a painful condition, is prevalent in the general population yet no program has been developed which can successfully treat this disorder in all cases. Recent evidence has shown that pain may be a limiting factor in a person's ability to rehabilitate their Achilles tendon. In order to help researchers develop a more effective rehabilitation program for tendinopathies, we will investigate the effects of topical diclofenac on individuals with CAT.

2. Background

Chronic Achilles tendinopathy (CAT) is a clinical condition characterized by a breakdown of collagen fibres, increased vasculature, and an increase of non-collagenous matrix in the Achilles tendon.[2] The Achilles tendon is an amalgamation of the gastrocnemius and soleus which inserts at the calcaneus and is the largest tendon in the body. Chronic Achilles tendinopathy often occurs as an overuse injury due to a failure in the body's healing response following repetitive tendon overloading. CAT is a common injury in athletes that participate in sports that involve running and jumping but the condition has also been found in overweight sedentary middle-aged adults. The cause and pathogenesis of chronic Achilles tendinopathy are likely to be diverse and multifactorial in many cases. Mafi et al. (2001) believes that age, sex, body composition, and ankle instability are predisposing factors that increase the odds of developing the chronic condition.[3]

The main symptom associated with chronic Achilles tendinopathy is pain within the mid portion or insertion site of the tendon.[3] Other symptoms include impaired performance, stiffness, and swelling.[4] Dissimilar to acute Achilles tendinopathy, there is much less inflammation or biochemical inflammation mediator, prostaglandin E_2, present within the Achilles tendon.[5] Recent studies have shown that there may be some inflammation in the connective tissue surrounding the tendon.[6] The exact mechanisms that cause the pain associated with CAT remain uncertain. Initially, it was hypothesized that the pain was due to an inflammation-repair response to overuse injury of the Achilles tendon. Alfredson et al. (1999) used a micro-dialysis technique to compare the biochemical concentrations of prostaglandin E_2 in normal tendons to

chronic tendinopathic Achilles tendons. The authors found that there was no significant difference between the two, although there was a non-significant trend for the injured tendon to display increased prostagladin E_2 levels. They concluded that the pain associated with CAT was not due to inflammation. During the same study, Alfredson et al (1999) found that the excitatory neurotransmitter glutamate was present in higher concentrations within the tendinopathic tendons. Glutamate is known to play a role in both central and peripheral pain regulation with nociceptors, and diclofenac is known to inhibit peripheral glutamate-mediated nociception.[25] Webbron (2008) uses the term neoneuralization to describe the proliferation of sensory nerve endings in response to pro-inflammatory cytokines in damaged tissue. He found that along with the new innervation, substance P, a neurotransmitter that is involved with the transmission of pain within the central nervous system, was much more abundant in tendinopathic tendons when compare to healthy tendons. In a study of rotator cuff disease, the amount of pain was moderately correlated with the amount of substance P in the subacromial bursa.[1] This indicates that substance P may also play a role in pain mechanisms. Diclofenac has recently been shown to inhibit substance P release from sensory neurons.[26] Thus, in addition to reducing the levels of classic inflammatory substances like progstagladin E_2 within the painful tendon, diclofenac could inhibit other relevant nociceptive mechanisms.

Exercise, in addition to its ability to maintain healthy tissue, can be used as a treatment for tissue repair and adaptation. Mechanotransduction is the process in which a cellular signal cascade is created in response to mechanical stimuli and promotes structural change within those or surrounding cells. This process occurs during exercise.[7] Fahlstrom et al. (2003) found that an eccentric calf training program done twice a day for 12-weeks at the maximum tolerable intensity (Alfredson protocol)[27] was clinically significant in reducing the symptoms in subjects with CAT. Eccentric exercise may increase tendon tensile strength and volume by altering the production of type 1 collagen.[8] Heavy, painful eccentric exercise has produced clinically significant results for rehabilitation although it is less effective in non-athletes and older adults.[7,19] Silbernagel et. al (2007) found that there were no negative effects from allowing individual with CAT to continue Achilles tendon loading activities (i.e. sports) while using a pain-monitoring model to guide their level of therapeutic exercise and recreational activities.[18,20] Thus, exercise appears to be a safe treatment for people with tendinopathy, who are mainly limited by the experience of pain.

Diclofenac is a non-steroidal anti-inflammatory drug (NSAID) that works by preventing an enzyme, cyclooxygenase (COX), from making prostaglandins.[15] Diclofenac also has analgesic effects which are the topic of ongoing investigations.[25] At high tissue concentration diclofenac can block sodium channels and prevent nociceptive afferent fibers from firing action potentials.[23] Diclofenac has been shown to minimize the pain associated with osteoarthritis and chronic lateral epicondylitis.[22] A Cochrane review written by Pattanittum et. al (2013) evaluated 5 studies that focused on the comparison NSAIDs and placebos on elbow tendon pain due to tendinopathy. The review concluded that topical NSAIDs were more effective than the placebo for short time periods (up to 4 weeks) but questioned the methodological rigour of the studies

performed. A crossover randomized study performed by Burnham et al (1998) evaluated the effectiveness of topical diclofenac for chronic lateral epicondylitis. He found that with the use of topical diclofenac (3 times a day for a week) subjects had a significant reduction of pain and wrist extensor weakness. Many studies have investigated the use of diclofenac with chronic elbow tendinopathy, however there is no current literature to our knowledge that investigates the use of diclofenac with chronic Achilles tendinopathy. Our own group recently conducted a pilot study (short-term, cross-over placebo controlled trial) which found that 10% diclofenac significantly reduced the pain associated with Achilles tendinopathy, and improved people's ability to generate force through the Achilles tendon.

2.1 Significance

This study can help contribute to the development of a possible adjunct treatment to reduce pain and improve Achilles tendon function among individuals with CAT.

3. Aims of Study

1. To determine whether a 4-week course of 10% topical diclofenac improves clinical outcome (VISA-A score) relative to placebo.

2. To determine whether any improvements seen with diclofenac compared to placebo persist 8 weeks later (i.e. 12 weeks from baseline)

4. Objective

The objective of this study is to evaluate the effects of topical diclofenac on the pain and impairment (VISA-A score) associated with chronic Achilles tendinopathy.

5. Hypothesis

Individuals treated with topical diclofenac will have a greater improvement in VISA-A score at 4 weeks than those treated with placebo gel.

6. Study Design

Placebo-controlled trial, random allocation in a 1:1 ratio.

7. Study Location

This study will be conducted at Fortius Sport and Health, Burnaby, Vancouver, Alan McGavin Sports Medicine UBC, and Dr. Bovard's practice (1200 Lonsdale Ave, North Vancouver).

8. Study Population

One hundred Vancouver community dwelling participants will be recruited from sporting clubs, running groups, gyms, community centres, running trails, and the Fortius and Alan McGavin Sports Medicine Centres and Dr Bovard's practice by health professionals' recommendations and posters.

9. Eligibility Criteria

Inclusion Criteria

1. Male and female subjects aged 19 years and older.

2. Fluent in English.

3. Subjects previously diagnosed with Achilles tendinopathy by a health care professional (imaging at the discretion of the treating physician) and demonstrating the following criteria – localized tendon pain and thickening, worsened with palpation and tendon loading activities, and no clinical suspicion of other diagnoses.

4. Symptoms for 3 months or more.

5. Subjects who are able to give informed consent.

6. VISA-A score less than 80.

7. Subjects who are engaged in an active rehabilitation program prescribed by an exercise specialist

Exclusion Criteria

1. Male and female subjects aged 18 years and younger.

2. Subjects with a BMI greater than 30.0.

3. Subjects with previous Achilles tendon rupture (complete or partial).

5. Subjects diagnosed with chronic pain syndrome, diabetes, hyperprproteinemia, metabolic syndrome, or systemic inflammatory diseases.

6. Subjects with a history of gastric ulcers, kidney disease or unstable hypertension.

7. Subjects with symptomatic osteoarthritis of the spine or lower extremities.

8. Subjects who have received corticosteroid injections in the Achilles region.

9. Subjects who take non-steroidal anti-inflammatory medication regularly.

10. Subjects who have been prescribed anticoagulants or fluroquinolones within the past 5 months.

11. Subjects with allergies to diclofenac or placebo cream.

12. Subjects who are unable to give informed consent.

10. Study Outcomes

10.1 Primary Outcome

VISA-A score (0-100) at 4 weeks.

10.2 Secondary Outcomes

1. Pain (Numeric Pain Rating Scale, 0 - 10)
i. Average pain during all tendon loading activities over last week
ii. Average pain during rehabilitative exercise over last week
iii. Average pain at rest during last week
iv. Current resting pain

2. Patient-reported change in symptoms (7 point scale from substantially better to substantially worse)

3. Pressure pain threshold (N) and transverse tendon stiffness (N/m) measured over the site of maximum tendon stiffness

11. Study Procedures

11.1 Recruitment and Screening

Subjects will be recruited by placing posters in sporting clubs, running groups, gyms, community centres, running trails, and medical clinics, or by running advertisements in local newspapers or websites. Once contacted, a telephone or email screen will be conducted to review minimal eligbility criteria and explain the purpose of the study. They will be advised that participants should receive no new treatment throughout the study, and to not make any changes to treatment they might already be receiving. If minimal criteria are met and the participant is interested in joining the study, a consent form will be emailed and a first study visit will be booked. The potential participants will be instructed to not do any moderate to vigorous physical activity 72 hours prior to Visit 1, and to refrain from taking any NSAIDS for 1 week (no analgesics for 24hrs).

Visit 1 – Week 0

Consent will be obtained by Erin Bussin. EB will complete the eligibility screen, which includes data collection (demographic information, BMI, rehabilitation history, and medical questionnaire including date of last NSAID taken). Subjects will complete the VISA-A questionnaire (a self-report questionnaire which measures Achilles tendinopathy severity). The investigator will use the MyotonPro to assess the transverse tendon stiffness at the location of maximal tendon thickening, and will use the AlgoMed Algometer to assess the subject's pain pressure threshold (PPT) on the most affected area of the Achilles tendon. Testing will be conducted at a controlled rate (30 KPa/s) with the subject lying prone on a treatment plinth. Pressure is gradually applied until the subject first experiences onset of pain, at which point they push a button. In rare cases where the person with CAT has baseline (resting) pain, then they will be instructed to press the button at the first increase in pain.

Subjects will then be issued with either placebo or 10% diclofenac gel. The gel will be supplied by a pharmacist, and the investigator and subject will not know the identity of the tubes. Subjects will be instructed to massage 1 gm of gel on the most painful area of the tendon for 30 - 45 seconds. The subject will also be instructed to how often to apply the gel (every 8 hours/3 times a day for 4 weeks) before the next scheduled appointment. They will be asked to complete a medication administration diary to confirm their compliance with the regimen, to document any medical or paramedical treatments received, and an exercise diary to record their exercise and physical activities.

Check-ins

The participants will be contacted twice by phone or email during weeks 1 and 3. The investigator will inquire about tolerability of the treatment and any side-effects, and remind the participant to keep filling out the diaries.

Visit 2

The second appointment will be scheduled 4 weeks following the initial appointment (within a 5 day margin of error). At the start of the second visit, subjects will be asked which treatment they thought they received, any remaining gel and the study diaries will be collected from the subject, and the outcome measures will be taken (VISA-A, pain, global improvement, non-protocol treatments, side effects, pain pressure threshold, tendon stiffness, returned to pre-injury activity level with no or minimal pain?).

Final follow-up

At 12 weeks from baseline (within a 5 day margin of error), participants will be contacted and the VISA-A administered by email. They will be asked the question about return to activity level again (returned to pre-injury activity level with no or minimal pain?). Subjects who received the placebo will be offered a 4-weeks supply of topical diclofenac.

11.3 Coding

All subject information (questionnaires, medical information, test results) will be coded to protect the identity of each subject. The subjects code ID will consist of a sex designation and two-digit code (e.g. M/F-##). The subject's information will be locked in a secure cabinet at Fortius Sport and Health. The subject's ID code and the subject information kept on computer files will be secured with a password. Co-investigators will be the only people with access to the locked cabinet and computer password.

11.4 Research tools used

For all measurements, in people with bilateral Achilles tendinopathy the worse side will be measured.

AlgoMed Algometer

The Algometer is a computerized pressure meter used to assess tissue pain perception in muscle pain syndromes. The device is set to a specific pressure (KPa) and placed on the site of maximal Achilles tendon pain. The subject's response to pressure threshold and tolerance tests are then processed by software and graphed on the computer for analysis. The subject will lie face down on a treatment plinth for the PPT measurement. The subject presses a button the moment they first begin to perceive pain, and the test is stopped and the pressure value recorded. The same site will be tested on both occasions.

MyotonPro

The MyotonPro is a hand-held device which imparts a small controlled impact (tap) to the surface of the Achilles tendon – the ensuing vibrations are measured by an accelerometer, and tissue stiffness in the transverse plane (N/m) is derived from this.

11.5 Safety Considerations

Topical Diclofenac

Side-effects to diclofenac are rare if administered correctly. A meta-analysis that evaluated the safety profile of topical diclofenac reported that diclofenac has a relatively high topical safety index especially when compared to the intolerance experienced with oral dosing.[24] Dry skin and rash were the most common local skin reactions reported in 9.0% and 3.0% of subjects, respectively, in placebo/diclofenac controlled studies.[24] The dry skin and rash were reported as mild-to-moderate and self-resolving.[24]

Physiologic Measurements

The two measures (pain pressure, tissue stiffness) have previously been reviewed by UBC CREB and are considered to be minimal risk, in that they do not expose subjects to any risk beyond what they would typically encounter in everyday life.

Pain pressure – this test involves the gradual application of pressure to the painful site, and the test is immediately stopped when the subject reports onset of pain. This is essentially an objectified equivalent of a person gently squeezing a painful spot to localize their pain. Based on clinical experience and reasoning, this type of test is not enough to cause injury – the application of gentle pressure to a painful site is standard practice in clinical examinations.

Tendon stiffness – this test applies a force of several newtons (about the weight of a strawberry).

12. Statistical Considerations and Data Analysis

The pain pressure threshold measurement will be repeated three times and the two closest measures will be used for analysis. To avoid wind up, we will wait one minute between each measure.

A repeat measures ANOVA will be used to detect the effect of time (0, 4, 12 weeks) or treatment (placebo vs active) on VISA-A score. Paired t-tests will be used to detect changes in pain, pain pressure threshold and tendon stiffness from baseline to 4 weeks. The proportion of individuals who have returned to full activity at the different timepoints will be reported as point estimates with 95% confidence intervals. The global rating of change (Likert scale) will be compared between groups using a Mann Whitney-U test.

<u>Sample Size Calculation</u>

We estimate that after 4 weeks, the placebo group will experience a clinically insignificant mean improvement of 5 (SD17) points on the VISA-A, compared to a clinically significant improvemnet of 15 (SD17) with diclofenac. For a two-sided t-test with alpha 0.05 and power 0.80, the required sample size is 46 per group.

13. References

1. Gotoh, M., Hamada, K., Yamakawa, H., Inoue, A., and Fukuda, H. "Increased Substance P in Subacromial Bursa and Shoulder Pain in Rotator Cuff Diseases" *Journal of Orthopaedic Research* (1998)16:618-621

2. Longo, U., Ronga, M., and Maffuli, N. "Achilles Tendinopathy" *Sports Medicine Arthroscopy Review* (2009)17:112-126.

3. Mafi, N., Lorentzon, R., and Alfredson, H., "Superior short-term results with eccentric calf muscle training compared to concentric training in a randomized prospective multicenter study on patients with chronic Achilles tendinosis" *Knee Surgery, Sports Traumatology, Arthroscopy* (2001)9:42-47.

4. Maffulli, N., Khan, K,. and Puddu, G. "Overuse Tendon Conditions: Time to Change a Confusing Terminology" *The Journal of Arthroscopic and Related Surgery* (1998)14: 840-843

5. Paavola, M., Kannus, P., Jarvinen, T., Khan, K., Joza, L., and Jarvinen, M. "Current Concepts Review: Achilles Tendinopathy" *Journal of Bone and Joint surgery* (2002)84: 2062 – 2076

6. Rees, J., Stride, M., and Scott, A. "Tendons – time to revisit inflammation" *British Journal of Sports Medicine* (2013)

7. Silbernagel, K. "Does One Size Fit All When It Comes to Exercise Treatment For Achilles Tendinpathy?" *Journal of Orthopaedics & Sports Physical Therapy* (2014)2:42-44.

8. Wilson, F., Bleakley, C., Bennett, K., and Mockler, D. "Exercise orthoses and splinting for Achilles tendinopathy (protocol)" *The Cochrane Library* (2013)12

9. Jensen, M., and MacFarland, C. "Increasing the reliability and validity of pain intensity measurement in chronic pain patients". *Pain* (1993)55: 195-203

10. Robinson, J., Cook, J., Purdam, C., Visentini, P., Ross, J., Maffulli, N., Taunton, J., and Khan, K. "The VISA-A questionnaire: a valid and reliable index of the clinical severity of Achilles tendinopathy" *British Journal of Sport Medicine*. (2001)5:335-41

11. Briggs, K., Lysholm, J., Tegner, Y., Rodkey, W., Kocher, M., and Steadman, J. "The Reliability, Validity and Responsiveness of the Lysholm and Tegner Activity Scale for Anterior Cruciate Ligament Injuries of the Knee: 25 Years Later. *American Journal of Sports Medicine* (2009)5:890-7

12. Alfredson, H., Thorsen, K., and Lorentzon, R. " In situ microdialysis in tendon tissue: high levels of glutamate, but not prostaglandin E_2 in chronic Achilles tendon pain". *Knee Surgery, Sports Traumatology, Arthroscopy* (1999)7:378-381

13. Fahlstrom, Martin., Jonsson, Per., Lorentzon, Ronny., Alfredson, Hakan. "Chronic Achilles tendon pain treated with eccentric calf-muscle training". *Knee Surg Sports Traumatol Athrosc* (2003)11: 327-333

14. Gammaitoni, A., Goitz, H., Marsh, S., Marriott, T., and Gler, B. "Heated diclofenac/tetracaine patch for treatment of patellar tendinopathy pain" *Journal of Pain Research* (2013) 6:565

15. Pattanittum, P., Turner, T., Green, S., and Buchbinder, R. "Non-steroidal anti-inflammatory drugs (NSAIDs) for treating lateral elbow pain in adults (Review)". *The Cochrane Library* (2013)5

16. Webborn, A. "Novel approaches to tendinopathy". *Disability and Rehabilitation (*2008) 30:1572-1577

17. Thomeé R. "A comprehensive treatment approach for patellofemoral pain syndrome in young women". *Journal of the American Physical Therapy Association and de Fysiotheraput.* (1997) 77:1690-1703.

18. Silbernagel,K., Thomee, R., Eriksson, B., Karlsson, J. "Continued Sports Activity, Using a Pain-monitoring Model, During Rehabilitation in Patients with Achilles Tendinopathy" *The American Journal of Sports Medicine* (2007)35:879-906

19. Sayana, K., Maffulli N. "Eccentric calf muscle training in non-athletic patients with Achilles tendinopathy." *Journal of Science and Medicine in Sport* (2007)10:52-58.

20. Silbernagel,K., Gustavsson, A., Thomee, R., Karlsson, J. "Evaluation of lower leg function in patients with Achilles tendinopathy" *Knee Surergy Sports Traumatol Arthroscopy* (2006)

21. Burnham, R., Gregg, R., Healy, P.,Steadward, R. "Effectiveness of Topical Diclofenac for Lateral epicondylitis" *Clinical Journal of Sports Medicine* (1998)8:78-81

22. Zacher, J., Altman, R., Bellamy, N., Bruhlamnn, P., Da Silva, J., Huskisson, E, Taylor, R. "Topical diclofenac and its role in pain and inflammation: an evidence-based review" *Current Medical Research and Opinion* (2008)24:925-950

23. Nair, B., Taylor-Gjevre, R. "A Review of Topical Diclofenac Use in Musculoskeletal Disease" *Pharmaceuticals* (2010)3:1892-1908

24. Taylor, R., Fotopoulos, G., Maibach, H. "Review – A safety profile of topical diclofenac: a meta-analysis of blinded, randomized controlled trials on muscloskeletal conditions" *Current Medical Research and Opinion* (2011)3:605-622

25. Dong, X., Svensson, P., Cairns, B. "The analgesic action of topical diclofenac may be mediated through peripheral NMDA receptor antagonism" *Pain* (2009) 147:36-45

26. Vellani, V., Franchi, A., Prandin, M., Moretti, S., Castelli, M.,Giacomoni, C., Sacerdote, P. "Effects of NSAIDs and paracetamol (acetaminophen) on protein kinase C epsilon translocation and substance P synthesis and releasein cultured sensory neurons" *Journal of Pain Research* (2013)6:111-120

27. Alfredson, H., Pietila, T., Johnsson, P., Lorentzon,R."Heavy-Load Eccentric Calf Muscle Training For the Treatment of Chronic Achilles Tendinosis" *The American Journal of Sports Medicine* (1998)26:360-366

28. Matheson, L., Duffy, S., Maroof, A., Gibbons, R., Duffy, C., Roth, J. "Intra- and inter-rated reliability of jumping mechanography muscle function assessments" *Journal of Musculoskeletal Neuronal Interaction* (2013)13:480-486
