# Supplementary material for: Topical diclofenac vs placebo for the treatment of chronic Achilles tendinopathy: A randomized controlled clinical trial
Source: PLoS One. 2021 Mar 4;16(3):e0247663. doi: 10.1371/journal.pone.0247663 (PMC7932128; doi:10.1371/journal.pone.0247663)
Supplement: Attachment — Submitted filename: PLOS article.docx [file pone.0247663.s006.docx]

Thank you for the opportunity to review this paper. On the whole it is well written, but I think there are some further points for consideration. Please see below.

The participants are not representative of the wider Achilles tendinopathy population; they are recruited from running clubs and sports clubs and were excluded if they had any commonly associated disease, such as a metabolic disorder. Not surprisingly they were all very active with low pain scores and high VISA-A scores. I think the title would benefit from changing to reflect this. This should also be considered in the discussion and conclusion.

The reporting of participant characteristics should be in line with recent recommendations: <https://bjsm.bmj.com/content/54/11/627>. Missing characteristics should be considered in the limitations section

Method used to generate random allocation sequence would benefit from justification. Why was this process used and not a more usual approach e.g block randomisation.

How sample size was determined would benefit from justification. Why a repeated Anova is used when the primary outcome is between group differences is not clear. This could leave the study underpowered and thus not able to detect a difference – 32 per group is a small number.

Clinical trials should include a measure for *each of the* *nine* *core domains* at a minimum, so that future meta-analyses will be able to better estimate treatment effects. <https://bjsm.bmj.com/content/54/8/444>. Missing domains should be discussed and considered in the limitations section

The clinometric limitations of the VISA-A should be discussed. <https://journals.sagepub.com/doi/pdf/10.1177/1071100718816953?casa_token=QBSv-wQQ9h8AAAAA:I5KUyJK4cGnFG-CQytrhvpbc4faxF2gHs1qg2j8aobFpGmhIIQ16HO8y5uDERpaqBrItufDt0K7KoyI>

<https://bjsm.bmj.com/content/52/19/1221.abstract>

Given the high levels oof scoring, could the tool be no be sensitive enough to change?

Line 145 is repeated and should be deleted

There are two full stops at the end of the last sentence on p.6.
