# Supplementary material for: Topical diclofenac vs placebo for the treatment of chronic Achilles tendinopathy: A randomized controlled clinical trial
Source: PLoS One. 2021 Mar 4;16(3):e0247663. doi: 10.1371/journal.pone.0247663 (PMC7932128; doi:10.1371/journal.pone.0247663)
Supplement: Attachment — Submitted filename: response to reviews.docx [file pone.0247663.s007.docx]

| **Reviewer comment** | **Response** |
| --- | --- |
| Indicate if the distribution of the data was checked for normality prior to applying the ANOVA or t-tests. | Thank you for the opportunity to verify this. In the process of checking this comment, we have found that data were normally distributed with the exception of the change in pressure pain threshold, which was right skewed. We have therefore adjusted this from a t-test to a Mann-Whitney U, however this does not alter the conclusion of the manuscript. Please see amended text in the statistical methods section. |
| Table 2: Indicate the percentages which correspond to the frequencies in Table 2. | The % for the frequency data (Sex, gradual onset, and location) has been added as requested |
| The participants are not representative of the wider Achilles tendinopathy population; they are recruited from running clubs and sports clubs and were excluded if they had any commonly associated disease, such as a metabolic disorder. Not surprisingly they were all very active with low pain scores and high VISA-A scores. I think the title would benefit from changing to reflect this. This should also be considered in the discussion and conclusion. | Thank you for this comment. We have been able to include this in the discussion and conclusion; but we’ve struggled with how to include this in the title because there were some physically inactive and overweight individuals in the sample so it’s hard to give it a clear-cut title pertaining to this issue. At the present time, we have left the title as is, but we are open to editorial suggestions.  The following text was added in the discussion: “The recruited sample were, on average, physically active, and did not demonstrate commonly associated disease such as a metabolic disorder.” And in the conclusion: “In conclusion, this study demonstrates a lack of clinical response to 4 weeks of treatment with either diclofenac or placebo, in healthy people with Achilles tendinopathy and no comorbidities.” |
| The reporting of participant characteristics should be in line with recent recommendations: <https://bjsm.bmj.com/content/54/11/627>. Missing characteristics should be considered in the limitations section | Thank you. We have reviewed the recommendations, and added a note that BMI was measured by us, included height and weight, and also added this statement to the limitations section on missing characteristics:  A limitation of this study is that we did not record information on the use of loading tests or diagnostic imaging, or whether there was prior history (e.g. previous episodes) of tendinopathy at the same or other locations, as suggested by new guidelines [24]. |
| How sample size was determined would benefit from justification. Why a repeated Anova is used when the primary outcome is between group differences is not clear. This could leave the study under-powered and thus not able to detect a difference – 32 per group is a small number. | Thank you for this opportunity to expand on this. As stated in the manuscript, we conducted a power analysis using G*power (v 3.1) for a repeat-measures ANOVA with two groups and three timepoints, including testing for a within-between group interaction. For the main outcome measure (VISA-A), we assumed an effect size for diclofenac of F=0.25 (medium), α=0.05, 1-β =0.95, correlation among repeated measures of 0.65, and ε=1. This yielded a sample size of 32 per group. A repeat-measures ANOVA was chosen because the primary outcome (VISA-A) was measured at 0, 4 and 12 weeks. In theory, this is a more powerful design than t-test, as we have multiple measures on which to compare the groups. This can be illustrated by conducting a sample size calculation for a simple t-test on the same data. The pooled standard deviation of the change score was 19 at week 4 and 20 at week 12. Assuming the MCID of the VISA-A score is 12 points (Cohen’s d of 0.63), to detect an increase over and above the placebo of 12 points at a single time poin would require a sample size of 44 people per group. So, the reviewer is correct in that 32 is a smaller number of required participants for the ANOVA design for a medium effect size. However, we definitely do acknowledge that this is a relatively small sample size for a clinical study. We have added this limitation in the discussion section:  “The participant population sample was a relatively small group of individuals…”  And we are open to other editorial suggestions on how to handle this issue. |
| Clinical trials should include a measure for each of the nine core domains at a minimum, so that future meta-analyses will be able to better estimate treatment effects. <https://bjsm.bmj.com/content/54/8/444>. Missing domains should be discussed and considered in the limitations section | We have added the following statement:  “A further limitation is that a newly released consensus statement has indicated that tendinopathy clinical trials should include outcome measures from each of 9 domains, and we neglected to measure 2 of the domains (psychological factors and quality of life.” |
| The clinometric limitations of the VISA-A should be discussed. <https://journals.sagepub.com/doi/pdf/10.1177/1071100718816953?casa_token=QBSv-wQQ9h8AAAAA:I5KUyJK4cGnFG-CQytrhvpbc4faxF2gHs1qg2j8aobFpGmhIIQ16HO8y5uDERpaqBrItufDt0K7KoyI> <https://bjsm.bmj.com/content/52/19/1221.abstract> Given the high levels of scoring, could the tool be no be sensitive enough to change? | We doubt that the lack of change in VISA-A score is due to a ceiling effect, because other studies with similar baseline VISA-A scores have demonstrated significant improvements with exercise.  Herrington L, McCulloch R. The role of eccentric training in the management of  Achilles tendinopathy: a pilot study. PhysTher Sport 2007;8:191–6.  Tumilty S, Mani R, Baxter GD. Photobiomodulation and eccentric exercise for Achilles tendinopathy: a randomized controlled trial. Lasers Med Sci 2016;31:127–35.  The starting VISA-A scores in our study are similar to those reported by the developers of the VISA-A in non-surgical populations.  Robinson JM, Cook JL, Purdam C, et al. The VISA-A questionnaire: a valid and reliable index of the clinical severity of Achilles tendinopathy. Br J Sports Med 2001;35:335–41. |
| Line 145 is repeated and should be deleted  There are two full stops at the end of the last sentence on p.6. | Thanks very much! |
| One inclusion criterion is that the patient must be "currently or previously engaged in a rehabilitation programme". I don't see the relevance of this to the research question. Furthermore, as you point out in the discussion, the nature of the involvement (or not) in a rehabilitation programme served as a confounding factor for the findings. I think the paper would benefit from a clearer justification for this issue, as well as further commentary in the discussion. | Thank you for the opportunity to elaborate on this point. We have addressed this in the methods and discussion as follows:  “According to our understanding of Canadian guidelines for ethical research, it is a requirement that participants must have received a standard level of care, therefore we required that participants be currently or previously engaged in a rehabilitation program for their Achilles problem.”  Discussion: “One of the inclusion criteria was that participants had to already have engaged in, or be currently engaged in, a rehabilitation program; we required this because we felt it was unethical to enroll patients who had not received a basic level of care for their condition. However, because assessing the effects of rehabilitation was not our primary goal, we did not document the details of, or compliance with, rehabilitation, and surprisingly only a minority of participants reported continuing with their rehabilitation programs during this study.” |
| L120. Concerning the "potential side-effects of diclofenac", as a non-medical practitioner I would value a summary of the key side-effects that might be relevant to this drug and as a science-reader, would like to know which side-effects the participants were informed of and by what basis participants were excluded should they occur (as was the case on occasion). | Thanks again – we have added this detail:  “Participants were told about the potential side effects of topical diclofenac (gastrointestinal upset or pain, heartburn, headache, dizziness, drowsiness, rash) and instructed to contact the study coordinator if they thought they were experiencing a side effect. In the case of a side effect, the principal investigator discussed the case with the attending physician (TG) who made the decision to exclude the patient or not. “ |
| L133. Table 1; NPRS is included within the Table title. I think it belongs as a table footnote (where you have placed the VISA-A abbreviation (L134)). Furthermore, you haven't provided an expansion for the abbreviation NPRS. This should be rectified. | Thank you – this has been done now. |
| L137. I don't understand this statement. We are in the methods section, and I read this as you describing results, yet with no detail. Please address this to allay confusion. | Thank you – this is a bit awkward, it’s item 6b of the Consort checklist.  We’ve rephrased it:  The outcome measures below were collected, as planned prior to the start of the trial: … |
| L145. The VISA-A was completed in person and then via email at a later date. My understanding of the VISA-A literature is that its validity across these different media has not been established. This may seem a little pedantic but in addition to that, my personal experience in executing this questionnaire leads me to believe that it might be possible that potentially significant sampling error may be introduced by this. I don't know if it would be sufficient to change the overall study findings, but I feel compelled to point this out and recommend you mention it in the discussion. | Thank you – we agree, this could introduce an unknown source of variability into the VISA-A measure. We have added this point in the discussion:  “It should also be noted that the recording of VISA-A was done in person (on paper) at weeks 0 and 4, but by email at week 12: this variation in VISA-A measurement may have influenced the results.” |
| L145. This sentence effectively repeats the sentiment of Lines 142-4. Suggest removal. | Thanks! This has been fixed. |
| L194. I am curious regarding the genesis of the "unplanned analyses". Can you please provide a more thorough justification for this? As you describe and then discuss these findings they seem reasonable, but in order not to appear as though you were fishing, the nature of unplanned/post-hoc analyses should be described with more clarity. | Thanks again. We have added the following sentences:  “We would like to emphasize that these analyses are not meant as a fishing expedition or to in any way water down the primary analysis of VISA-A, but rather provide additional supplementary information not intended to alter the conclusions of the planned analysis:”  …  And: “The genesis of these analyses was from collaborative discussions among the co-authors attempts to provide additional insight into the study’s results, particularly the lower-than-anticipated participation in rehabilitation, and the unexpected trend toward worse outcome measures in women.” |
| L213. Table 2; can you confirm that there was no difference in symptom duration between the diclofenac and placebo groups? | We have now confirmed this with the added text  (Table 2: no significant differences). |
| L222 (& L239, L249, L250, L256). I would like to see evidence from you statistical testing articulated here (eg p values). It would be best approached I think as a Table. | We have provided the requested p-values in the text. |
| L249. "...non-significant trend for PPT to be higher at 4 weeks." Given significance testing (for all its faults), provides a binary outcome. I don't think it is appropriate to articulate such findings in this manner; it speaks of bias. Please remove. | Thanks, we have removed this statement. |
| L305. In your opening discussion paragraph I think value would be added by summarising your key findings, including p-values. | Thanks very much – we do lead with the key finding “This study demonstrated that there is a lack of clinical response to 4 weeks of treatment with topical diclofenac compared with placebo, in people with Achilles tendinopathy” and would rather focus the opening discussion paragraph on the clinical response rather than the p-values. |
| L338. You describe the Fredberg study, but have not provided any relevance to the inclusion of it in your discussion. ie you do not allude to their findings and certainly do not describe them in the context of your study. Recommend revising this or removing altogether. | We have removed reference to the Fredberg study. |
| L358. Can you provide reference(s) for the estimates of MCID. To my knowledge there is less validation of MCID than you imply here. Please clarify. | Absolutely – this issue has been reviewed recently so we are able to cite a paper in which MCID estimates have been compared and discussed.:  Murphy M, Rio E, Debenham J, et al. Evaluating the progress of mid-portion Achilles tendinopathy during rehabilitation: A review of outcome measures for self-reported pain and function. Int J Sports Phys Ther 2018;13:283-292. |
